# A brief history of and future prospects for pneumococcal vaccination in Malaysia

**DOI:** 10.1186/s41479-023-00114-8

**Published:** 2023-08-25

**Authors:** Alex J. J. Lister, Evelin Dombay, David W. Cleary, Lokman H. Sulaiman, Stuart C. Clarke

**Affiliations:** 1 Faculty of Medicine, Institute for Life Sciences, University of Southampton, Southampton, UK; 2 Institute of Microbiology and Infection, College of Medical and Dental Sciences, University of Birmingham, Birmingham, UK; 3 NIHR Birmingham Biomedical Research Centre, Institute of Translational Medicine, Birmingham, Birmingham, UK; 4 Centre for Environment and Population Health, Institute for Research, Development, and Innovation, International Medical University, Kuala Lumpur, Malaysia; 5 Department of Community Medicine, School of Medicine, International Medical University, Kuala Lumpur, Malaysia; 6 NIHR Southampton Biomedical Research Centre, University Hospital Southampton NHS Trust, Southampton, UK; 7 Global Health Research Institute, University of Southampton, Southampton, UK; 8 School of Postgraduate Studies, International Medical University, Kuala Lumpur, Malaysia; 9 Centre for Translational Research, Institute for Research, Development, and Innovation, International Medical University, Kuala Lumpur, Malaysia

**Keywords:** Pneumococcal disease, Pneumococcal vaccination, Malaysia, Vaccination policy, Pneumonia

## Abstract

Pneumococcal pneumonia remains a significant global public health issue. Malaysia has recently added the 10 valent pneumococcal conjugate vaccine to its national immunisation programme. Data on pneumococcal serotype epidemiology is vital for informing national vaccination policy. However, there remains a lack of representative population-based pneumococcal surveillance in Malaysia to help both the assessment of vaccine effectiveness in the country and to shape future vaccine policy. This review explores the history of pneumococcal vaccination, the burden of pneumococcal disease in Malaysia, and offers an insight into the prospects for reducing pneumococcal disease in Malaysia.

## Pneumococcal disease and vaccination

Pneumonia remains one of the biggest killers globally and is a leading cause of infant mortality, particularly in South Asia and Western Pacific regions [1]. One of the major aetiological agents of pneumonia, *Streptococcus pneumoniae*, has the potential to cause various diseases ranging from the more serious infections including septicaemia, meningitis and severe pneumonia, to milder diseases such as acute otitis media thereby posing a significant public health burden. For example, approximately 80–90% of children up to the age of 5 years suffer from acute otitis media which becomes the leading cause of paediatric healthcare visits and a frequent cause of antibiotic prescriptions, with pneumococci isolated from between 20 and 50% of presentations [2–4]. Importantly, the bacterium can reside in the nasopharynx of healthy populations, which is known as carriage, but it can also cross mucosal membranes and stimulate a large inflammatory reaction through the cytokine cascade in the host [5].

There are more than 100 different serotypes of *S. pneumoniae* [6] which are identified through the composition of the polysaccharide capsule on the surface of the bacterium. Not all serotypes cause severe disease, and they can differ in their colonisation frequency, ability to invade the host, their antibiotic resistance profile, and epidemiology [7–9].

There are several licensed vaccines for use against pneumococcal disease, which stimulate an epitope-specific immune response to the pneumococcal polysaccharide contained in the vaccine. This results in the recognition and immune protection against vaccine serotypes. The first pneumococcal vaccines became available around a hundred years ago, whereby miners were inoculated with various doses of killed pneumococci after a study of pneumococcal pneumonia in that population [10]. There are now two types of pneumococcal vaccine available on the market, pneumococcal polysaccharide vaccine (PPV) and pneumococcal conjugate vaccine (PCV), with differing serotypes in each formulation (Table 1). PPV is recommended for those aged over 65 years and those with underlying health conditions, however, the specific recommendations for PPV23 may vary depending on the country and the guidelines issued by national health authorities or organizations. PCVs, on the other hand, can be used in both adults and children, although they have thus far been mostly used in young children, as they have the advantage of eliciting a T-cell-mediated immune response which leads to immunological memory [11, 12]. Prevnar® (PCV7, Pfizer) was the first PCV available on the market and provided protection against seven pneumococcal serotypes. It has since been superseded by higher valency vaccines such as Synflorix® (PCV10, GlaxoSmithKline) and Prevnar 13® (PCV13, Pfizer). Licensed in 2009 and 2010 respectively these protect against 10 and 13 serotypes. Other vaccines have been developed including the 15 serotype Vaxneuvance (Merck Sharp & Dohme Corp.) and 20 serotype Apexxnar (Pfizer, Inc.), both of which have recently been approved by the European Medicines Agency and the Food and Drug Administration for use in those aged ≥ 6 weeks and > 18 years of age respectively [13–15].

**Table 1:**
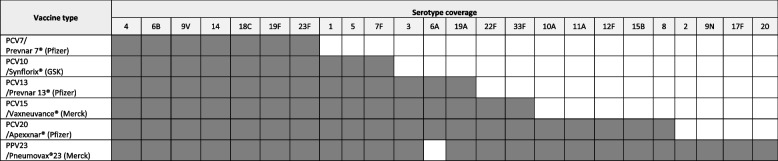
Serotypes included in licensed pneumococcal vaccines. PCV20 covers the largest number of serotypes and includes those serotypes already included in lower valency PCV’s. PPV23 covers all serotypes included in PCV20 except 6 A, but additionally includes serotypes 2, 9 N, 17 F, 20 not covered by any currently licensed PCV’s

The development of PCV’s has been hailed as a major benefit to global health, with an estimated 7 million deaths prevented by 2030 [16]. Despite the evidence highlighting the benefit of pneumococcal vaccination, only 51% of the global infant population has received their final dose, with only 19% in the World Health Organisation (WHO) Western Pacific Region. In addition, by the end of 2021, 46 of the 194 WHO member countries had still to add PCV to their national immunisation programmes [17, 18]. Without routine immunisation, pneumococcal disease will continue to cause morbidity and mortality globally.

## Pneumococcal disease burden in Malaysia

**Fig. 1:**
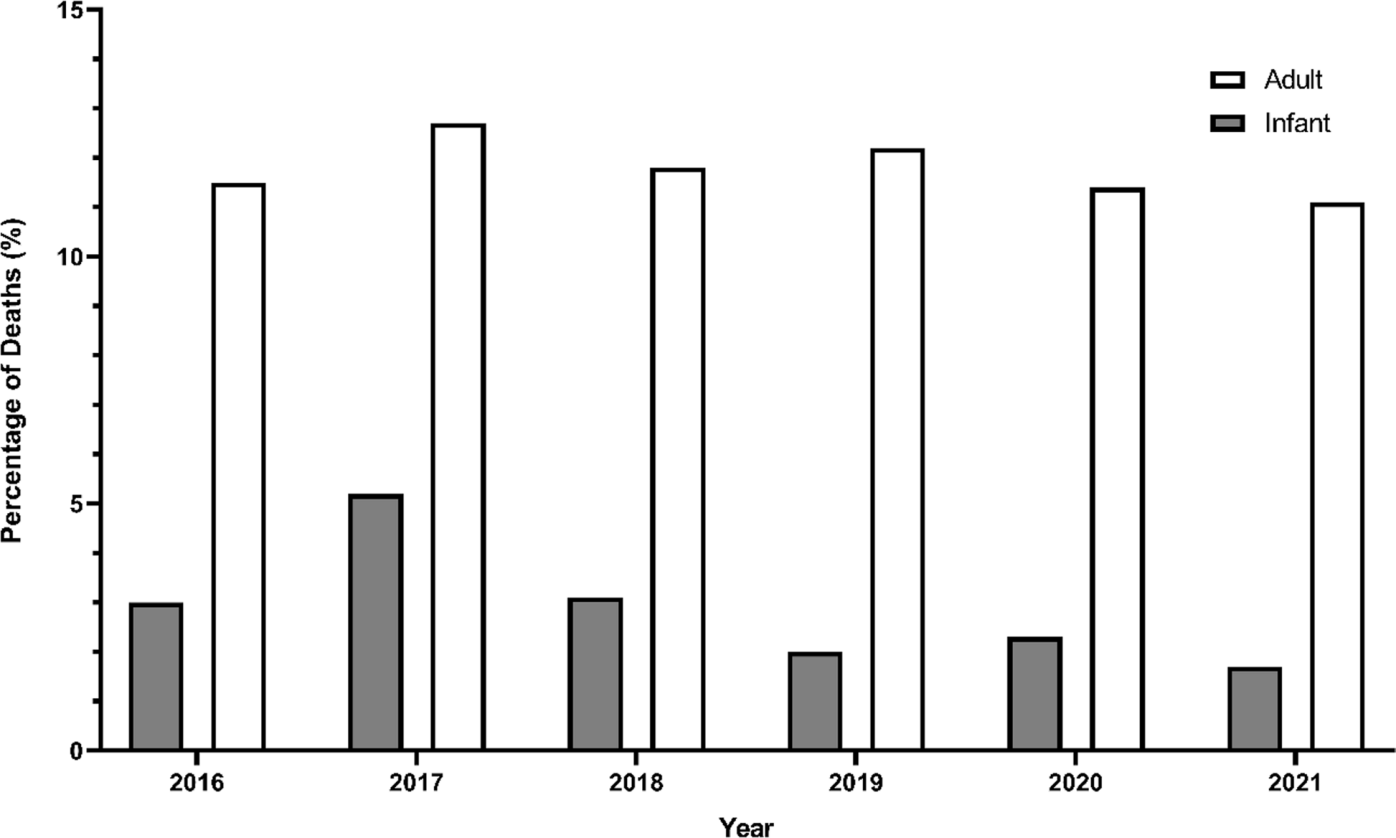
Pneumonia deaths as a percentage of total causes of death 2016–2021 in adults and infants. Adult deaths have remained relatively constant at just over 10%, whereas infant deaths have remained around 3%. Data from Department of Statistics Malaysia [21]

Malaysia has a population of over 32 million and is one of the wealthiest and most developed countries in the WHO Western Pacific Region. It is classified by the World Bank as an upper middle-income country and is expected to achieve high income status between 2024 and 2028. However, there are some reforms needed to facilitate this, including improvement to basic health and nutrition. Malnutrition has been associated, for example, with an increased risk of severe childhood diseases such as pneumonia [19]. According to the National Health Morbidity Survey conducted by the Institute for Public Health Malaysia in 2019, 14.1% (95% CI: 11.39, 17.37) of children below 5 years of age were classed as underweight and 21.8% (95% CI: 18.42, 25.63) of children below 5 years were classed as stunted [20]. In addition, malnutrition and stunting were found to be more prevalent in children living in rural areas [20]. Moreover, the Department of Statistics Malaysia reported that, in 2021, pneumonia was the third highest cause of death (11.1%) following COVID-19 (19.8%) and ischaemic heart disease (13.7%) (Fig. 1). Pneumonia is the third highest (10.8%) and second highest (11.5%) cause of death in men and women respectively [21].

It remains unclear why, relative to other causes of death, Malaysia has a high burden and mortality of pneumonia although it has been shown that early cessation of breastfeeding, exposure to passive smoking and availability of pharmaceutical interventions contribute to worse outcomes in children with pneumonia in Malaysia [22].

## National immunisation programme

The oversight of health systems, policy and infrastructure is the responsibility of the Ministry of Health as a federal government body. In collaboration with the Malaysian Paediatric Association and the Malaysian Society of Infectious Diseases and Chemotherapy, the National Immunisation Programme is strongly supported by the Immunise4Life initiative, an expert-driven community that promotes immunisation of people of all ages.

Developed in the 1950s, Malaysia’s national immunisation programme (NIP) was based on the expanded programme on immunisation from the WHO and now includes vaccines against thirteen preventable diseases [23]. Despite the high burden of pneumonia in the region, Malaysia was not eligible for support from GAVI, the Vaccine Alliance, due to its upper middle-income status [24]. It was only at the end of 2020 that Malaysia added PCV10 to its NIP in a three-dose schedule.

Prior to the inclusion of PCV10 in the NIP, PCV’s were available in Malaysia from around 2005, with private health care facilities offering PCV7, and then PCV10 or PCV13, to those who could afford and access the vaccine [25]. During the time of PCV being available privately, there was poor coverage in the population, made worse due to the lack of health education for Malaysian parents about the availability and importance of pneumococcal vaccination [26]. It is estimated that between 15 and 18% of the total yearly birth cohort receives private doses of the vaccine, which equates to roughly 450,000 to 500,000 children [27]. To aid the education around vaccines a programme was set up by the Malaysian Paediatric Association which aimed to increase knowledge and awareness on maternal, child and family care [28]. The ‘YES to Pneumococcal Protection!’ campaign supported several public awareness initiatives to help educate the public about vaccination and awareness about pneumococcal disease [29].

The commitment to nationwide pneumococcal vaccination for children was initially included as a political pledge by the Alliance of Hope (Pakatan Harapan) in their manifesto during the 14th General Election in 2018 as a statement of “*The Pakatan Harapan Government will also provide compulsory pneumococcal vaccination for all children under the age of 2 years, as is the practice in many other countries*” [30]. This commitment was fulfilled in 2020 when a budget of RM60 million (~ 13.4 million USD) was allocated to its implementation, with an aim of completion by June 2020 [31]. However, with the COVID-19 pandemic, this was delayed until December 2020 [32]. A cost-benefit analysis by Shafie et al. in 2019 [33] provided some estimates of the vaccine acquisition and concluded that over a 5-year time horizon, the implementation of PCV13 would be more cost-effective. However, it is important to emphasize that the ultimate decision regarding the choice between PCV10 and PCV13 depends on the preferences of the decision-maker or policymaker. It is crucial to take into account factors such as affordability and sustainability when considering a national vaccination program. Consequently, based on the report’s findings, it was suggested that administering PCV13 to high-risk groups initially, followed by potential implementation for all children under 5 years old, may be a prudent course of action. This consideration likely influenced the final decision to implement PCV10 in the National Immunization Program (NIP) [34].

Following the introduction of PCV, the reported uptake of the vaccine between January and September 2021 was 95% for the first dose, and just slightly lower at 93% for the second which is much higher than the original 71% target [35]. Malaysia has historically had a good track record for vaccination uptake, although vaccine hesitancy has increased in recent years, so the education of parents on the importance of their children receiving immunisations on schedule is important for public health [36]. Since the COVID-19 pandemic caused major disruption globally, and as the pandemic was ongoing when PCV was implemented in Malaysia, there might still be problems for parents and guardians accessing health services for children to receive immunisation. This is likely to be more problematic in rural areas of Malaysia where distances to health services are increased. Around 95% of the urban Malaysian population live within 5 km to the nearest public health centre and 95.6% within 5 km to a private health centre. A smaller percentage is seen in the rural population, where 82.4% live within 5 km to a public health centre and 55.1% to a private facility [37].

Unlike privately available vaccines, those included in the NIP are free for children, except foreigners. The Malaysian government is committed to ensuring high coverage is reached, and that there is a strong surveillance and monitoring system to assess disease epidemiology [38]. This includes an online immunisation registry that is to be launched to gather and analyse vaccination data more effectively, with further aims to identify the lack of vaccine compliance and potential low-vaccine-uptake communities. This initiative is supported by the recently made recognition that since the COVID-19 pandemic, anti-vaccination movements may be acting as a barrier to the aims of the NIP and future improvements. However, a study on the Selayang community showed most participants accept and understand the benefits of pneumococcal vaccination which is positive [39].

## Health technology assessment

In 2011, a consensus was reached among paediatricians from both public and private sectors to include PCV in the National Childhood Immunization program due to its high efficacy. However, the Committee for Vaccine Use and Cost has proposed a further examination of the cost-effectiveness of introducing PCV into the National Immunization Program.

Hence, in 2014, to assess the efficacy, safety, effectiveness, cost-effectiveness, and organizational aspects of PCV10 and PCV13, a Health Technology Assessment (HTA) was conducted prior to its inclusion in the National Childhood Immunization program. The HTA report on child immunisation commented on the 23 valent polysaccharide vaccines being poor at generating immune responses in children for the protection against pneumococcal disease [34]. The Committee for Vaccine Use and Cost requested that further study on the cost effectiveness of PCV should be undertaken prior to its inclusion in the NIP and it was then that a second report was published outlining the views on the need for national PCV introduction [40]. It stated that there was no policy regarding pneumococcal routine vaccination. The report ultimately did not provide the much-needed guidance to the health practitioners and did not use evidence readily available on the recommendation to include PCV in the NIP. This resulted in delays when pneumococcal vaccination was not accessible to the general population. If more data on pneumococcal epidemiology and cost-effectiveness had been available, then PCV introduction might have been approved earlier [41].

## Present policy

In accordance with the NIP, childhood immunisation with PCV10 follows a three-dose schedule, at four months, six months and fifteen months of age for those children born on or after the 1st January 2020, with an adjusted schedule for children already above the age of five months. Despite the risk of infection in the elderly population, pneumococcal polysaccharide vaccination has not been added to the NIP but is recommended by the Malaysian Society of Infectious Diseases and Chemotherapy [42]. It is recommended that all adults at 60 years or above receive one dose of PCV13 at least one year after any previous dose of PPV23 [43]. These recommendations include the use of PCV13 in adults with immunocompromising conditions and therefore a high risk of IPD.

Notably, it has also been stated that priority should be given to infant coverage of conjugate vaccination which in turn will provide herd immunity effects to the older populations [43]. It was also concluded that the addition of adult pneumococcal vaccination to the NIP would have a large financial implication, which was also a barrier to the implementation of childhood PCV in the country for many years with a range of RM 132 million to RM 241 million calculated for PPV implementation based on an uptake of 70% in the population [43].

## Disease surveillance

Due to the high prevalence of asymptomatic colonisation of *S. pneumoniae* in young children, which is a prerequisite for the development of disease, the surveillance of serotype epidemiology is deemed essential. Like many other countries, the list of notifiable diseases under the Prevention and Control of Infectious Diseases Act 1988 (Act 342) in Malaysia does not explicitly include pneumococcal disease. Surveillance of *S. pneumoniae* in Malaysia is undertaken through a combination of approaches through the Ministry of Health, such as studies conducted through the Institute for Medical Research and the National Public Health Laboratory. Following the introduction of pneumococcal vaccination, pneumococcal pneumonia should be included in Schedule 1 of the Prevention and Control of Infectious Diseases Act (Act 342), which lists other infectious diseases which are endemic. It is through this act where main surveillance mechanisms are employed.

As mentioned previously, there are few disease and carriage studies conducted in other areas outside of the capital Kuala Lumpur, and many available studies were from hospitals or medical centres, which carry limitations such as risk of data duplication and difficulty in defining populations [44]. There are some academic institutions across Malaysia which have associated private healthcare facilities, which do not contribute to the national surveillance programmes, thus limiting surveillance efforts. Additionally, surveillance can be hampered when it comes to referring isolates to the National Public Health Laboratory and the Institute of Medical Research from rural communities. As serotype-specific data from IPD cases is considered the gold standard for the measurement of the effectiveness of pneumococcal vaccination programmes, the lack of a robust laboratory surveillance system poses a challenge to obtaining a true representation of vaccine effectiveness [45]. Vaccine evasion by *S. pneumoniae* has been observed following vaccine rollout and it has been linked to the alteration of genetic arrangement within lineages expressing various capsular types which plays an important role in serotype replacement [46, 47]. Notably, Global Pneumococcal Sequence Clusters (GPSCs) associated with various serotypes including both vaccine (VT) and non-vaccine-types (NVTs) have a major potential to offset the effectiveness of pneumococcal vaccination. For example, GPSC10 has been revealed to express more than 16 serotypes amongst which only six are included in PCV13 [48], thus highlighting the need for continual surveillance.

Antimicrobial resistance is an increasing problem globally and the Malaysian government has formed an action plan to reduce the threat of antimicrobial resistance called MyAP-AMR. This focuses on four themes, including public education, surveillance and research, infection control and appropriate antimicrobial use [49]. In Malaysia, antibiotics are commonly prescribed for the treatment of upper respiratory tract infections [50]. According to a cross-sectional study from 2016, antibiotics were most frequently prescribed for URTIs (49.2%), and prescription rates were higher in private primary care settings compared to public hospitals in Malaysia suggesting that antibiotics might be often prescribed inconsistently and inappropriately [51], something that could be reduced following routine vaccination with PCV’s. A high prevalence of resistance to antibiotics has been seen in pneumococcal isolates from Malaysia, with multi-drug resistance commonly seen which is comparable to other Asian countries [52]. Pneumococcal vaccines have been shown to reduce antimicrobial-resistant serotypes in both vaccinated infants and unvaccinated adults through the reduction of circulating serotypes, thus highlighting the importance of effective childhood vaccination [53, 54].

## Future prospects and decisions

Now that PCV15 and PCV20 have been licensed for use [55, 56], those countries that have already or are yet to add PCV to their NIPs, will need to ascertain which vaccine best suits their healthcare needs. The CDC recommends that the routine immunisation of children with PCV13 or 15 in a four-dose schedule should be implemented and that children who have missed the routine doses should have catch-up doses dependent on the age at which vaccination starts [57]. The tender price is inclusive of catch-up vaccination among children < 5 years within the Malaysian PCV10 programme. However, at the time of writing, there have been no official statements from the Ministry of Health in Malaysia on the upgrading from PCV10 to higher valency conjugates.

We suggest that further carriage and disease studies are required to assess the effectiveness of the newly implemented PCV10 and to monitor serotype changes in the population. Until there is data available post-vaccine implementation, it will not be possible to assess the morbidity and mortality associated with pneumococcal infection nationally [41].

To the best of our knowledge, no publication has yet determined the relative cost-effectiveness of PCV15 nor PCV20 in Malaysia. Conjugate formulation vaccines have been effective against pneumococcal infections but are not without limitations with a high cost of production, the potential for serotype replacement, and lower efficacy against non-invasive disease. This has led to research into protein-based vaccines, based on the idea that they are not serotype specific and thereby should have greater serotype coverage [58, 59]. As PCV10 has only recently been introduced, it is more likely that a discussion on higher valency PCV’s will be had before alternatives are explored.

Increases in serotypes not covered by the 10-valent conjugate may result in the need to switch to higher-valency vaccines. Our recent review showed that before the introduction of PCV, the majority of serotypes most prevalent in Malaysia were included in PCV10, with serotype 19 F being the most common serotype across pooled data from invasive, non-invasive and carriage studies [44] and was again the most common serotype in both carriage and non-invasive studies. Serotype 6B was also identified as one of the most common serotypes. Previous epidemiological studies have shown high levels of antibiotic resistance in 19 F [60], which is concerning due to the prevalence of the serotype. The continued active surveillance into circulating serotypes is imperative, thus highlighting the need for large-scale studies into both carriage and disease.

## Conclusion

*S. pneumoniae* remains a leading cause of global morbidity and mortality in children and adults. Malaysia has made an important step towards reducing the burden of disease by adding PCV10 to its NIP. However, there is a need for improved surveillance to understand the impact of the vaccine on circulating pneumococci in both disease and carriage. Nationwide or sentinel carriage studies across Malaysia should be considered, ensuring representation of the whole population, which will assist with understanding the impact of PCV. With the field of pneumococcal vaccine research constantly evolving, Malaysia should also continually assess the potential for implementation of higher valency vaccines to help reduce the burden of pneumonia.

## Data Availability

Not applicable.
